# Study on interspecific relationships, community stability, and environmental factors of lithophytic moss at different elevations in karst cities

**DOI:** 10.1002/ece3.70120

**Published:** 2024-08-06

**Authors:** Lixin Duan, Xiurong Wang, Yalin Jin

**Affiliations:** ^1^ College of Forestry Guizhou University Guiyang Guizhou China

**Keywords:** bryophyte, environmental factor, interspecific relationship, karst city, plant diversity, stability

## Abstract

Ecosystem stability arises from the interplay of species diversity, environmental conditions, and external disturbances. Understanding the structure of plant communities, interspecific relationships, and community stability in urban ecosystems is fundamental to ecological restoration and community development. This study utilized the karst city of Guiyang as a case study and employed the *α* diversity index, variance ratio method (*VR*), *χ* ^2^‐test, Pearson correlation test, Spearman rank correlation test, M. Godron stability, and canonical correspondence analysis (CCA). The research focused on analyzing the species diversity, interspecific associations, community stability, and environmental factors of lithophytic moss at various elevations (989–1398 m). The findings revealed the presence of 58 species belonging to 27 genera and 13 families of lithophytic moss in the study area. Notably, the Brachytheciaceae and Pottiaceae emerged as dominant, exhibiting a broad ecological range and adaptation mechanisms, thereby playing a crucial role in the ecological environment of rocky desertification. The study observed that the highest species richness and dominance values of lithophytic moss were recorded at the N4 (1296–1398 m) elevation gradient, while the highest species diversity and uniformity values were observed at the N3 (1194–1295 m) elevation gradient, indicating a significant impact of altitude on lithobryophyte species diversity, particularly at middle and high altitudes. The analysis of interspecific associations and stability indicated a predominantly negative overall association within the lithophytic moss community, suggesting an early stage of succession, with weak interspecific associations and correlations among dominant pairs, tending towards relative independence. Only the communities at N2 (1092–1193 m) elevation exhibited stability, while the other communities were in an unstable stage, showing no significant correlation with species diversity. Furthermore, light intensity (182–129300 lux) exerted the greatest influence on community stability. Additionally, air humidity (36.5–52.3%) and altitude (998–1327 m) emerged as the primary environmental factors influencing community distribution, with a close and positive correlation between the two. These results hold significant reference value for promoting the succession and steady development of vegetation in rocky desertification areas and enhancing the conservation and restoration of vegetation community diversity in karst urban ecosystems.

## Graphical abstract

The study on the interspecific relationship, community stability, and environmental factors of lithophytic moss at different elevations in karst cities can improve the conservation and restoration of vegetation community diversity in karst urban ecosystems.

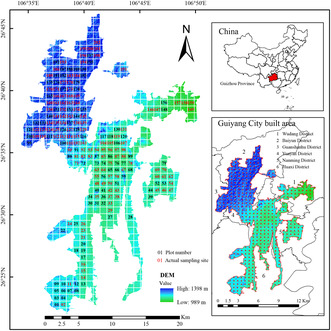

## 1 INTRODUCTION

The rapid decline in global biodiversity has elevated the relationship between species diversity and ecosystem stability, along with their underlying maintenance mechanisms, to a critical scientific issue (Mougi & Kondoh, 2012). Species diversity is essential for assessing the level and functional state of community organization, serving as a metric for the complexity of community structure and function (Wiegand et al., 2017). Interspecific associations elucidate the composition, structure, and stability of current plant communities, facilitating an understanding of species interactions and ecological relationships during community development (Chen et al., 2023; Legendre & Legendre, 1983). These associations are foundational to the formation and evolution of plant communities and play a significant role in promoting their stable development (Gu et al., 2017). Community stability and species diversity exhibit considerable variation across different successional stages, with community stability providing a direct indicator of the community's current successional state (Geng et al., 2022). Currently, the relationship between species diversity and ecosystem stability is not uniformly understood. Some scholars posit that community stability is positively and negatively correlated with species diversity (Campbell et al., 2011; She et al., 2023; Wang et al., 2022; Xu et al., 2021), while others argue that community stability is not correlated with species diversity (Giehl & Jarenkow, 2015; Lves & Carpenter, 2007). Therefore, further investigation is warranted to understand the mechanisms sustaining the relationship between community diversity and stability.

Community stability, as a comprehensive reflection of ecological succession, serves as a vital indicator of ecosystem sustainability (Wang et al., 2022). Ecosystem stability is influenced by numerous factors. Research has demonstrated that environmental variables, such as climate, terrain, and soil, significantly impact species composition, diversity, and community stability (Li et al., 2022; Wu et al., 2023). The interplay between environmental factors, diversity, and stability is highly intricate, arising not from a single factor but from the complex interactions among multiple elements (Kang et al., 2020). Investigating the spatial distribution of species along environmental gradients and its association with community stability is a central concern in plant community ecology. Some researchers have examined the influence of elevation on interspecific associations and community stability, revealing that elevational factors can alter the relationships between certain species pairs, interspecific cohesion, and community stability (Du et al., 2023). However, the implications for plant communities in karst regions remain ambiguous. Elucidating the connections between community stability, species diversity, and environmental factors is fundamental to constructing resilient and adaptable ecosystems.

Guiyang City, located in southwest China, is a principal region of karst ecosystem distribution. This area has long been afflicted by rocky desertification, leading to significant ecological and environmental challenges, including soil erosion, severe vegetation degradation, a marked decrease in biodiversity, and diminished community stability (Wen et al., 2024). In recent years, the restoration of vegetation has emerged as a critical issue in the management of damaged karst ecosystems. In areas affected by rocky desertification, plant stability is considered a key indicator of ecological recovery. Bryophytes, notably, play a pivotal role in the restoration of karst rocky desertified areas due to their soil stabilization, water retention, ecological restoration, and environmental monitoring capabilities (Rosentreter, 2020; Ruklani et al., 2021). Lithophytic mosses, as pioneers in the ecological restoration of karst rock surfaces, can expedite the dissolution of these surfaces by secreting organic acids and other substances, thereby fostering the formation of a biological microenvironment and promoting vegetation succession (Liu et al., 2017). There have been studies focusing on the restoration capabilities of lithophytic moss ecosystems in karst regions. These studies have examined aspects such as the relationship between soil and water retention, species diversity, and the distribution patterns and environmental interactions of lithophyte moss during the process of rocky desertification (Jin & Wang, 2023; Tu et al., 2021).

However, research on interspecies associations and community stability of lithophytic moss within urban ecosystems remains scarce. Current studies on interspecific interactions in karst regions have predominantly concentrated on trees, shrubs, herbs, and phytoplankton (Ma et al., 2021; Song et al., 2022; Yang et al., 2023; Yu et al., 2017). Findings suggest that in areas of intense rocky desertification, bryophytes are at an initial successional stage, resulting in highly unstable communities (Yin et al., 2016). In the Chongqing region and Baotianman Nature Reserve, moss communities are observed to be in the early to middle stages of succession, with most species existing independently rather than in association (Fan et al., 2018; Liu et al., 2019). Similarly, bryophyte species in the Tianshan No. 1 periglacial area exhibit strong autonomy and minimal interspecific reliance (Yuan et al., 2022). These studies collectively reveal that bryophyte communities undergo successional processes, predominantly in the early or middle stages. Nonetheless, the factors influencing the succession and stability of bryophyte communities across different regions have not been conclusively determined. Moreover, given that the community development cycle of bryophytes is significantly shorter than that of woody plants, they offer excellent models for testing theories of community succession (Fan et al., 2018). Consequently, elucidating the species composition and successional dynamics of lithophytic moss in karst urban environments can provide a theoretical foundation for the restoration and reconstruction of vegetation in areas affected by rocky desertification.

This study focused on the main urban area of Guiyang City, examining the composition, and diversity distribution patterns of lithophytic bryophyte communities, and their associated environmental factors across an altitudinal gradient, and community stability and interspecific association were combined to reveal the competition status, community structure and community succession trend of plant populations. The research aimed to address several key questions: (1) What are the compositional and diversity characteristics of lithophytic moss communities at different elevations within karst regions? (2) How do dominant lithophytic moss species interact across various altitudinal gradients? (3) What is the current successional stage of lithophytic moss communities in the study area? (4) Which factors are critical in influencing species distribution and community stability? The outcomes of this investigation are intended to provide a theoretical framework for understanding the community structure and interspecific relationships of lithophytic moss in karst urban environments in China, as well as foundational data to support the conservation and restoration of plant diversity in areas affected by rocky desertification.

## 2 MATERIALS AND METHODS

### 2.1 Study area

Guiyang City, situated in the heart of the Qianzhong Mountains (106°07′ – 107°17′ E, 26°11′ – 26°55’ N) on the Yunnan‐Guizhou Plateau in Southwest China, boasts a diverse topography dominated by karst formations. This region is characterized by its stark rocky desertification and extensive areas of bare rock. Elevations range from 880 to 1659 m, with the highest point in the southeast and the lowest in the central region. The city experiences a subtropical monsoon climate with a humid ambiance, evidenced by an annual average temperature of 15.3°C, relative humidity of 77%, total annual rainfall of 1215.7 mm, and 1162.6 h of sunshine per year (https://www.guiyang.gov.cn). Guiyang is endowed with abundant plant resources, bryophytes thrive within the urban environment, particularly lithophytic mosses that colonize rock faces and walls, displaying a remarkable variety of species.

### 2.2 Research methods

**FIGURE 1:**
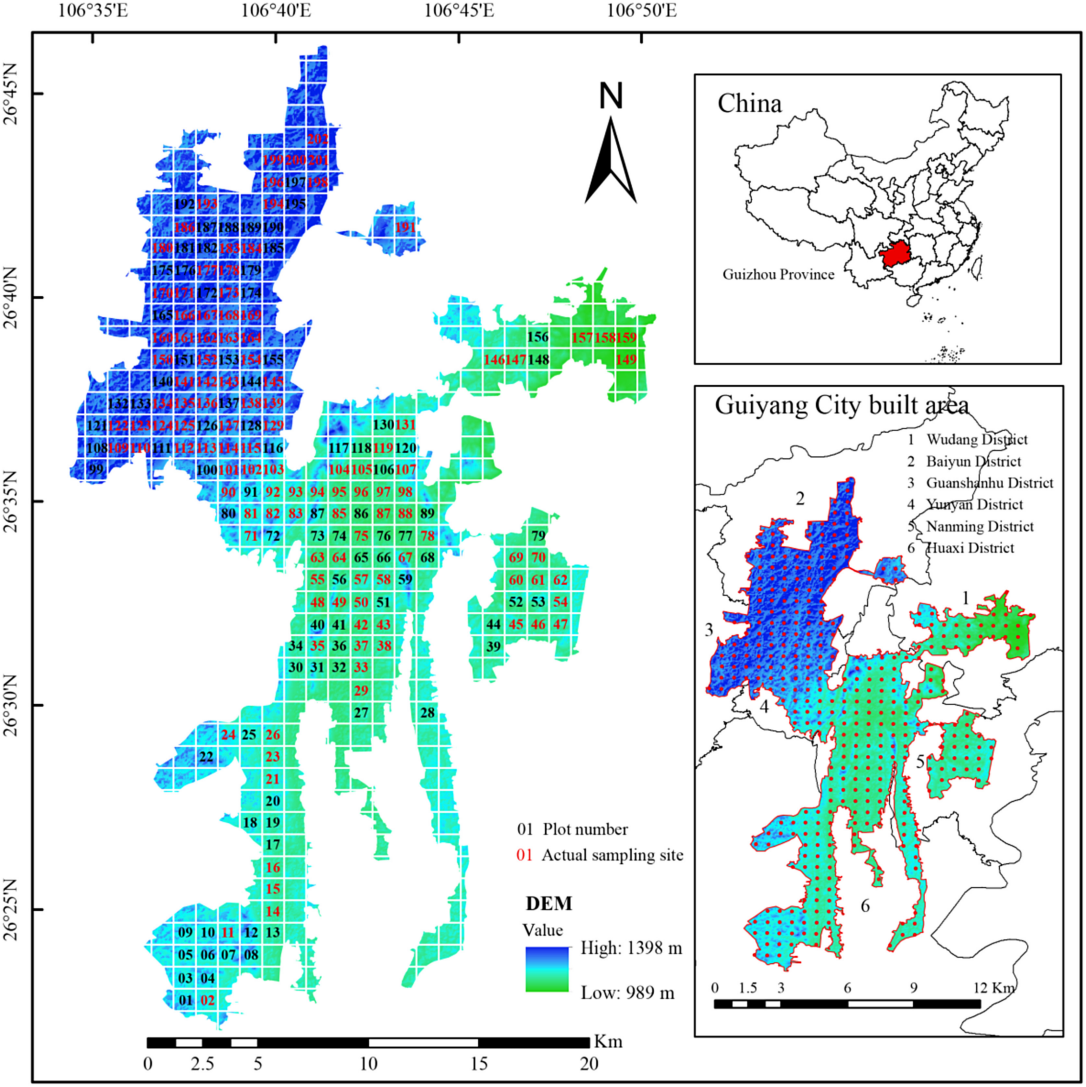
Setting of urban lithophytic moss community sample plot.

From March to May 2022, we surveyed lithophyte moss in the downtown area of Guiyang with a sample method (Nanming District, Yunyan District, Huaxi District, Wudang District, Baiyun District, and Guanshan Lake District). Geographical elevation data (DEM) for the study area was obtained from the geospatial data cloud (http://www.gscloud.cn/). The six central urban areas were divided into four altitude segments based on equal height difference intervals: N1 (989–1091 m), N2 (1092–1193 m), N3 (1194–1295 m), N4 (1296–1398 m), with the boundaries of urban center built‐up areas referenced from Sun (Sun et al., 2023). Although the study area covers a large area, large‐scale grids (ranging from 1 × 1 km to 3 × 3 km) were systematically utilized to extract different types of green patches in grid units (Jiang et al., 2021), enabling a more comprehensive analysis of plant community characteristics in different regions, as bryophytes are distributed in various habitats and spatial scales (Monteiro et al., 2023). Consequently, the grid method was employed to establish sample plots. Using the fishing net grid tool in ArcGIS 10.2 software with a 1 km interval between sample points, a total of 117 sample sites were investigated after excluding some inaccessible or non‐conforming sample sites (Figure 1). For each sampling plot, a 10 × 10 m large sample square was designated, which was further subdivided into five 2 × 2 m middle sample squares. Within each middle sample square, five 10 × 10 cm small sample squares were established using a metal grid, following the five‐point sampling technique (Jin & Wang, 2023). Bryophyte coverage and the number of specimens collected were documented for each quadrat, with the process replicated three times on each substrate type during the survey. In total, 427 bryophyte specimens were gathered and subsequently identified under a microscope in the laboratory (Xiong, 2011, 2014a, 2014b). The voucher specimens have been archived in the laboratory of the College of Forestry at Guizhou University in Guiyang City.

Nine environmental factors were considered in this study, comprising elevation, slope aspect, slope gradient, vegetation coverage, canopy density, light intensity, air temperature, air humidity, and rock exposure. Altitude and coordinates (latitude and longitude) were determined using GPS, while slope aspect and gradient were measured using geological compasses. Canopy density was assessed using the method outlined by Smith and Ramsay (Smith & Ramsay, 2018), and light intensity, air humidity, and temperature were recorded using a DLY 1802 illuminometer and an LM‐8000A meteorograph. Additionally, vegetation coverage was measured visually (Qin et al., 2006) as the percentage of the vertical projected area of vegetation within the unit area. Rock exposure was quantified following Ding's method (Ding & Zhou, 2019), which calculates the ratio of bare rock area without vegetation or soil cover to the total rock surface area.

### 2.3 Data analysis

#### 2.3.1 Importance value and species diversity

The importance value (*L*) was calculated using relative coverage and frequency to assess the relative significance of plant species within the community (Liu et al., 2008). The *α* species diversity at various elevations was quantitatively characterized by employing the Margalef richness index (*R*), Shannon–Wiener Diversity Index (*H*), Simpson Dominance Index (*D*), and Pielou evenness index (*J*) (Zhang, 2018).

(1) $$L=\left(\mathrm{MI}+\mathrm{NI}\right)/2$$

(2) $$R=\left(S-1\right)/\ln\;N$$

(3) $$H=-\sum_{i=1}^{s}P_{i}\ln P_{i}$$

(4) $$D=1-\sum {\mathrm{Pi}}^{2}$$

(5) $$J=H^{\prime }/\ln\;S$$

where *L* is the ecological importance value; MI is relative coverage; and NI is the relative frequency. Where *P*_*i*_ = *n*_*i*_/*N*, *N* is replaced by the total moss coverage, *n*_*i*_ is replaced by the coverage of the *i* species, *H* represents the Shannon–Wiener Diversity Index, and *S* represents the number of plot species.

#### 2.3.2 Tests for overall association and interspecies association

Schluter's (Schluter, 1984) variance ratio method (*VR*) was used to describe the overall connectedness of plant communities, and the significance of correlation coefficients was tested:

(6) $$\delta_{T}^{2}=\sum_{i=1}^{S}P_{i}\left(1-P_{i}\right)$$

(7) $$S_{T}^{2}=\left(\frac{1}{N}\right)\sum_{j=1}^{N}\left(T_{j}-t\right)$$

(8) $$\mathrm{VR}=S_{T}^{2}/\delta_{T}^{2}$$

(9) $$p_{i}=n_{i}/N$$

(10) W=N×VR

where *S* is the total number of species, *N* is the total number of quadrats, $T_{j}$ is the number of species in the *j* quadrat, $n_{i}$ is the number of species in the *i* quadrat, and *t* is the average number of species in the quadrat. When VR = 1, species are independent; otherwise, they are positively correlated (VR > 1) or negatively correlated (VR < 1). Statistical *W* is used to further test the degree of deviation between *VR* value and 1. When $\chi_{0.95,N}^{2}$ < *W* < $\chi_{0.05,N}^{2}$, there is no significant correlation between species. On the contrary, there is a significant correlation between species.

The Yates's correction formula Chi‐square test (*χ* ^2^) was used to calculate the interspecific association of the dominant species in the community (Pandey et al., 2023), which is the qualitative index of interspecific association:

(11) $$\chi^{2}=\frac{N{\left[\left(|,\mathrm{ad}-\mathrm{bc},|\right)-\left(1/2\right)N\right]}^{2}}{\left(a+b\right)\left(c+d\right)\left(a+c\right)\left(b+d\right)}$$

*N* is the quadrat number. When *ad* > *bc*, the interspecies pairs are positive, and when *ad* < *bc*, the interspecies pairs are negative. If 3.841 < *χ* ^2^ < 6.635, the association was significant, *χ* ^2^ ≥ 6.635 was extremely significant, otherwise, it was not significant. sp. assoc() in R language spaa package was used to calculate the population and interspecies association.

#### 2.3.3 Jaccard index

The *χ* ^2^‐test provides an accurate and objective measure of the significance of interspecies associations but does not quantify the strength of these associations. In contrast, the Ochiai index (OI), Dice index (DI), and Jaccard index (JI) are capable of expressing the magnitude of interspecies associations. Xing et al. (2007) found that variations in these indices were highly significant (*p* < .001). Any of these indices can independently indicate the degree of interspecific association. Consequently, this study employed the JI to quantify the probability and extent of association between species pairs:

(12) $$\mathrm{JI}=\frac{a}{a+b+c}$$

The JI range is [0, 1]. The larger the value is, the stronger the positive correlation is; otherwise, the closer the negative correlation is. The JI value is calculated using sp.assoc() in the R language spaa package.

#### 2.3.4 Interspecific correlation

To assess the degree of correlation between species, the Pearson correlation coefficient and the Spearman rank correlation coefficient were utilized (Zhang et al., 2022), which are quantitative indicators of interspecific correlation. This approach allows for a comprehensive enhancement of the *χ* ^2^ test.

(13) $$r_{s\left(i,k\right)}=\left[\sum_{j=1}^{N}\left(x_{\mathrm{ij}}-\bar{x_{i}}\right)\right]/\sqrt{\sum_{j=1}^{N}{\left(x_{\mathrm{ij}}-\bar{x_{i}}\right)}^{2}{\left(x_{\mathrm{kj}}-\bar{x_{k}}\right)}^{2}}$$

$r_{s\left(i,k\right)}$ is the Pearson correlation coefficient between species *i* and species *k* in the quadrat, *N* is the total number of quadrats, $x_{\mathrm{ij}}$ and $x_{\mathrm{kj}}$ are the important value of species *i* and species *k*, respectively. They form vector sums $\bar{x_{i}}$ and $\bar{x_{k}}$. $\bar{x_{i}}$ and $\bar{x_{k}}$ are the average values of the importance of species *i* and *i* in the *j*‐quadrat, respectively. The value of (0,1] $r_{s\left(i,k\right)}$ is positively correlated, the value of [−1, 0) $r_{s\left(i,k\right)}$ is negatively correlated, and the value of (0) $r_{s\left(i,k\right)}$ is not correlated.

Spearman rank correlation coefficient needs to convert the abundance vector into a rank vector, and then calculate the rank vector by substituting it into the formula. Spearman rank correlation coefficient is calculated as follows:

(14) $$r_{p\left(i,k\right)}=1-\left[6{\left(x_{\mathrm{ij}}-\bar{x_{i}}\right)}^{²}{\left(x_{\mathrm{kj}}-\bar{x_{k}}\right)}^{2}/N^{3}-N\right]$$

where, $r_{p\left(i,j\right)}$ is the Spearman rank correlation coefficient between species *i* and species *k* in the *j* quadrat, *N* is the total quadrat number, $x_{\mathrm{ij}}$ and $x_{\mathrm{kj}}$ is the rank of the abundance value of species *i* and species *k* in the *j* quadrat respectively.

Within the R language spaa package, the sp.pair() function was used to calculate the correlation coefficients, and the significance of these coefficients was tested using the corr.test() function from the psych package. Data visualization was conducted using OriginPro 9.0.

#### 2.3.5 Community stability

The M. Godron stability analysis method is a systematic and comprehensive mathematical ecological approach for assessing community stability, providing a reliable reflection of community development and change trends (Zhou et al., 2017). Utilizing the improved M. Godron stability determination method (Godron, 1972; Zheng, 2000), known as the contribution law method, a regression model was constructed based on community species and their occurrence frequency to infer changes in major species during community succession. The specific method involves gradually accumulating the relative frequency of different plant species in the sample plot in descending order, followed by the reciprocal of the total number of plant species (total bryophyte coverage was considered) and its gradual accumulation based on the order of plant species. Subsequently, the reciprocal percentage of plant species corresponds to the cumulative relative frequency. By taking the cumulative relative frequency as *y* and the cumulative species as *x*, a scatter smooth curve model with the formula *y* = *ax* ^2^ + *bx* + *c* is established, intersecting with the linear equation model *y* = 100−*x*. The community's stability level is higher when the intersection of the two lines is closer to the stable point (20.00, 80.00).

To examine the relationship between community stability and the community diversity index, both the Pearson correlation coefficient and the Spearman rank correlation coefficient were employed. Statistical analyses were performed using SPSS Statistics 25.0. To study the relationship between species composition, environmental factors, and community stability of plant communities at different elevations, Detrended correspondence analysis (DCA) in Canoco 5.0 was used to sort the distribution of plant species in the sample plots (Zhang, 1992). It overcame the arcuation effect based on correspondence analysis (CA) data of all species at various points. The results showed that the length of the ranking axis was >4. Therefore, canonical correspondence analysis (CCA) based on an unimodal model was used for direct ranking. For each scale, two matrices (Braak & Milauer, 2012) are created: one for “vegetation attributes” × “sample site” (response variable), and the other for “habitat variable” × “sample site” (explanatory variable). The ranking results objectively reflect the ecological relationship between plant communities and the environment. Through the selection and screening method mentioned above, only the factors that have a significant impact on species distribution are added to the final ranking model, and the model is tested by Monte Carlo.

## 3 RESULTS AND ANALYSIS

### 3.1 Species composition and diversity characteristics of community

#### 3.1.1 Species composition

In the karst urban survey plots, a total of 58 bryophyte species were identified, encompassing 13 families and 27 genera, as detailed in Table S1. The dominant families were Brachytheciaceae (21 species) and Pottiaceae (11 species). The dominant species were presented in Table 1. Species composition varied across altitude gradients in the following order: N4 (1296–1398 m), with 39 species across 10 families and 19 genera, was the most diverse, followed by N2 (1092–1193 m) (32 species, 9 families, 15 genera), N3 (1194–1295 m) (28 species, 8 families, 17 genera), and N1 (989–1091 m) (24 species, 6 families, 12 genera). At N4 (1296–1398 m), the predominant species were *Brachythecium salebrosum*, *B. coreanum*, *Haplocladium angustifolium* and *Didymodon ferrugineus*, with *Haplocladium angustifolium* being the most dominant across the entire community. These species are commonly found in karst regions.

**TABLE 1:** Important values of dominant species of urban stone bryophyte community (top 15 species).

Number	Species	Total importance value (L)	Importance value (L)			
			N1	N2	N3	N4
S1	*Haplocladium angustifolium* (Hampe et C Müll.) Broth	1.499	0.339	0.445	0.458	0.257
S2	*Brachythecium salebrosum* (Weber. & Mohr) Schimp	1.359	0.320	0.368	0.217	0.453
S3	*Didymodon ditrichoides* (Broth.) X. J. Li & S. He	1.343	0.195	0.371	0.418	0.359
S4	*Haplocladium microphyllum* (Hedw.) Broth	1.325	0.250	0.386	0.243	0.445
S5	*Weissia exserta* (Broth.) P. C. Chen	1.175	0.327	0.175	0.286	0.387
S6	*Brachythecium coreanum* Cardot	0.997	0.132	0.458	0.294	0.113
S7	*Hyophila involuta* (Hook.) Jaeg	0.970	0.230	0.213	0.228	0.300
S8	*Didymodon ferrugineus* (Schimp. ex Besch.) Hill	0.964	0.536	0.061	0.262	0.368
S9	*Bryum calophyllum* R. Br	0.928	0.290	0.141	0.434	0.063
S10	*Brachythecium amnicola* Müll. Hal	0.832	0.269	0.207	0.188	0.168
S11	*Bryum dichotomum* Hedw	0.794	0.290	0.233	0.183	0.089
S12	*Eurohypnum leptothallum* (C. Meull.) Ando	0.728	0.160	0.192	0.090	0.286
S13	*Claopodium aciculum* (Broth.) Broth	0.684	0.293	0.114	0.278	0.335
S14	*Brachythecium albicans* (Hedw.) Schimp	0.670	0.386	0.199	0.192	0.085
S15	*Taxiphyllum taxirameum* (Mitt.) Fleisch	0.668	0.288	0.219	0.161	0.129

*Note*: Elevation gradient: N1 (989–1091 m), N2 (1092–1193 m), N3 (1194–1295 m) and N4 (1296–1398 m).

#### 3.1.2 *α* species diversity

**FIGURE 2:**
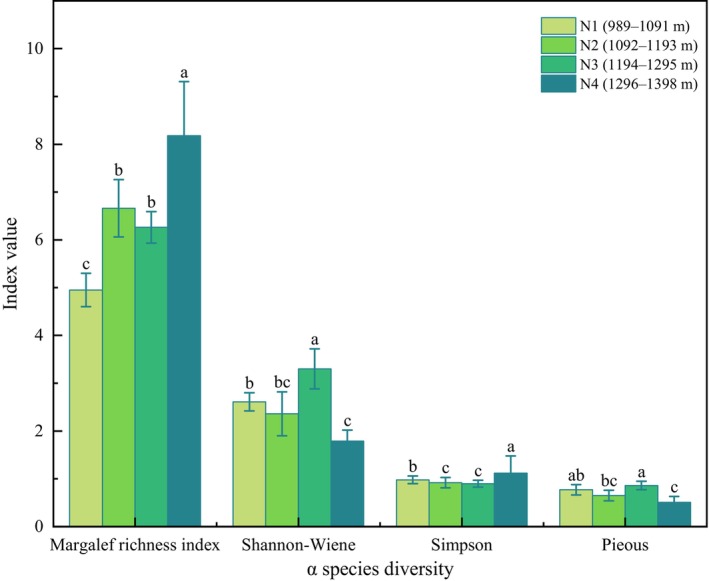
Analysis of lithophytic moss communities and *α* species diversity at different elevations. Different lowercase letters in the figure indicate that there are significant differences between the indexes of each altitude gradient (*p* < .05).

Species diversity serves as an indicator of community species richness, the distribution of species within the community, adaptation to physiographic conditions, and community stability (Zhong et al., 2019). Significant variations in the species diversity index were observed across different elevations (Figure 2). The highest Margalef richness index and Simpson dominance index were recorded at N4 (1296–1398 m), whereas the lowest values were found at N1 (989–1091 m) and N3 (1194–1295 m). Conversely, N4 exhibited the lowest Shannon–Wiener Diversity Index and Pielou evenness index, while N3 displayed the highest values. The *α* diversity at N2 (1092–1193 m) was intermediate, suggesting that species richness and diversity are greater at mid‐to‐high elevations.

### 3.2 Determination of interspecific associations

#### 3.2.1 Overall association analysis

The overall association (*VR*) of the dominant lithophytic moss communities was <1 (Table 2), and the test statistic (*W*) did not reach the threshold, signifying a significant negative correlation within the overall community. The *VR* values for the altitudinal ranges N1–N4 (989–1398 m) were 0.040, 0.043, 0.039, and 0.282, respectively. These results indicate that bryophyte communities across the four altitudinal gradients also exhibit a significant negative association. Consequently, the urban lithophytic moss community appears to be in an early successional stage and is highly susceptible to environmental influences. Even minor environmental fluctuations can result in compositional shifts within the bryophyte community.

**TABLE 2:** Overall association of dominant species of lithophytic moss at different elevations in urban areas.

Elevation (the number of plots)	Variance ratio (VR)	Statistic (*W*)	*χ* ^2^ ($\chi_{0.95,N}^{2}，\chi_{0.05,N}^{2}$)	Results
Totality (117)	0.143	16.73	[93.03, 143.25]	Significant negative correlation
N1 (24)	0.040	0.98	[13.85, 36.42]	Significant negative correlation
N2 (30)	0.043	1.30	[18.49, 43.77]	Significant negative correlation
N3 (17)	0.039	0.67	[8.67, 27.59]	Significant negative correlation
N4 (46)	0.282	12.98	[31.44, 62.83]	Significant negative correlation

*Note*: Elevation gradient: N1 (989–1091 m), N2 (1092–1193 m), N3 (1194–1295 m) and N4 (1296–1398 m).

#### 3.2.2 Relationship between species pairs of dominant species

**FIGURE 3:**
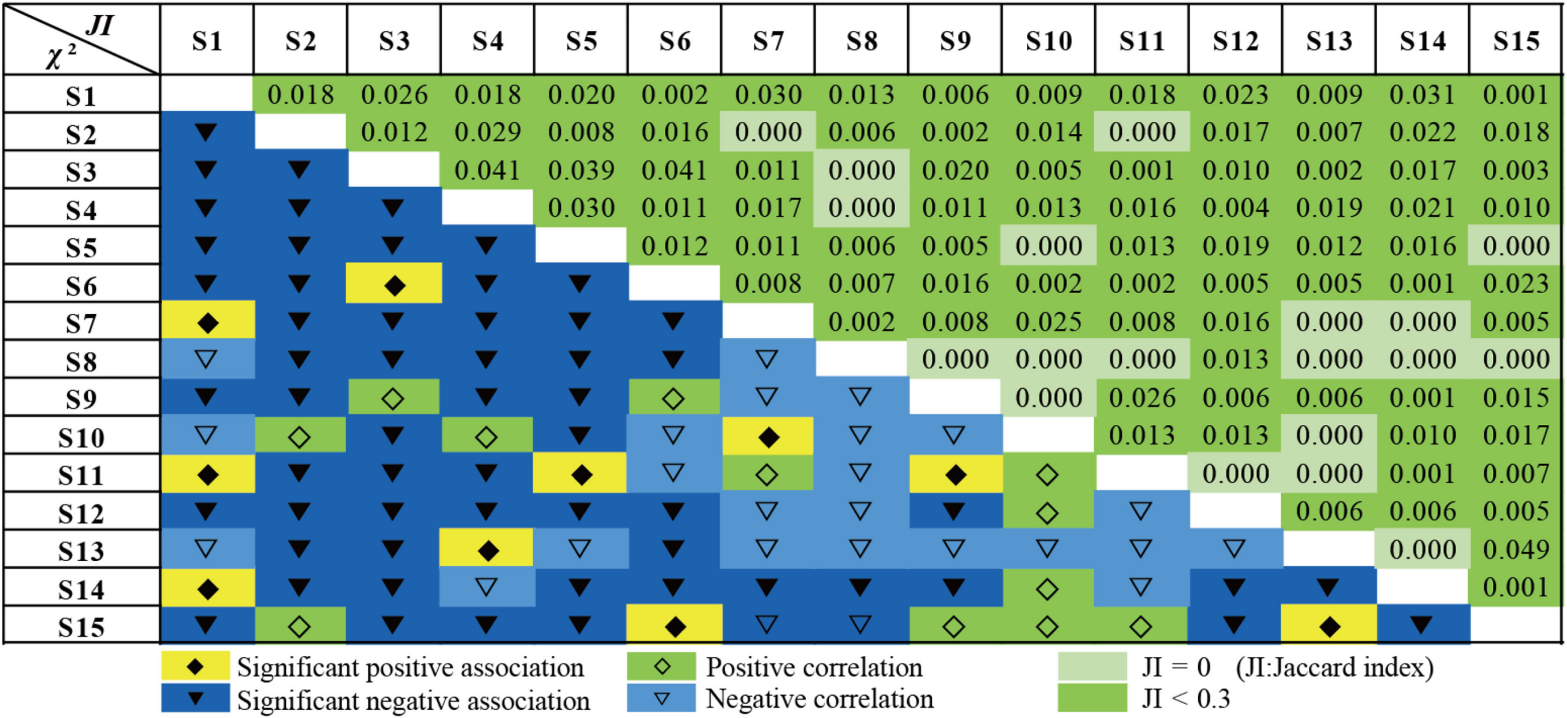
Chi‐square test of total dominant species and Jaccard index semi‐matrix. Species numbers are given in Table 1.

The Chi‐square (*χ* ^2^) test results, as presented in Table 3, Figure 3, and Table S2 revealed that the proportion of nonsignificant species pairs within the population and across the altitudinal communities N1–N4 (989–1398 m) were 35.24%, 1.00%, 99.05%, 100.00%, and 93.33%, respectively. These findings suggest a predominance of negative correlations among species pairs at different altitudinal gradients. Notably, the frequency of negatively associated species pairs exceeded that of positively associated pairs. This indicates a distinct ecological niche differentiation among the 15 bryophyte populations, with each species exhibiting unique ecological adaptability and a low incidence of co‐occurrence among species pairs.

**TABLE 3:** Chi‐square test and Jaccard index comparison of dominant species of lithophyte moss at different elevations in urban areas.

Elevation	Pair species number	*χ* ^2^‐test			Jaccard index		
		Positive association	Negative association	Positive and negative association ratio	JI ≥ 0.3	JI < 0.3	JI = 0
Totality	105	22 (20.95%)	83 (79.05%)	0.27	0 (0%)	86 (81.90%)	19 (18.10%)
N1	105	20 (19.05%)	85 (80.95%)	0.24	0 (0%)	51 (48.57%)	54 (51.43%)
N2	105	21 (20.00%)	84 (80.00%)	0.25	0 (0%)	45 (42.86%)	60 (57.14%)
N3	105	18 (17.14%)	87 (82.86%)	0.21	0 (0%)	44 (41.90%)	61 (58.10%)
N4	105	23 (21.90%)	82 (78.10%)	0.28	0 (0%)	43 (40.95%)	62 (59.05%)

*Note*: Elevation gradient: N1 (989–1091 m), N2 (1092–1193 m), N3 (1194–1295 m) and N4 (1296–1398 m).

Furthermore, the Jaccard index results, detailed in Table 3, Figure 3, and Table S2 demonstrated a clear dominance of species pairs with a JI <0 across the entire elevational range. Within the N1–N4 (989–1398 m) altitudinal gradient, species pairs with a JI of 0 were most prevalent, while no species pairs had a JI >0.3. These Jaccard index outcomes were in alignment with the *χ* ^2^‐test results, indicating a strong correlation with each dominant species at N1 (989–1091 m), whereas N2 (1092–1193 m), N3 (1194–1295 m) and N4 (1296–1398 m) exhibited weaker correlations. Overall, the inter‐species correlations within plant communities across different elevational gradients were found to be weak.

### 3.3 Determination of interspecies correlation

**FIGURE 4:**
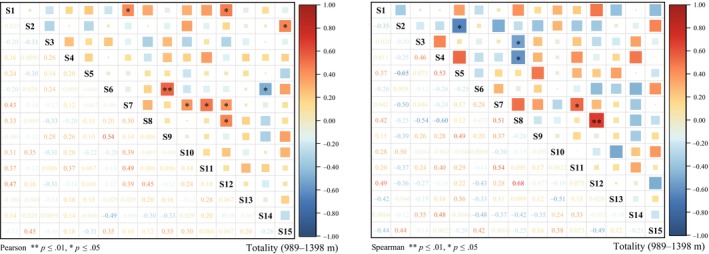
Semi‐matrix of Pearson and Spearman rank correlation test for dominant species with total altitude gradient. Species numbers are the same as in Table 1.

The Pearson correlation test results, as depicted in Table 4 and Figure 4, revealed that the ratios of positive to negative correlations for the population and altitudinal communities N1–N4 (989–1398 m) were 0.28, 0.24, 0.24, 0.21, and 0.28, respectively. The logarithmic frequency of negatively associated species was significantly greater than that of positively associated species, suggesting a generally weak relationship among most species. Furthermore, the occurrence of very significant and significant correlations among species pairs was infrequent, indicating a low level of interdependence between species. Despite some degree of association, species generally exhibited a relatively independent distribution pattern.

**TABLE 4:** Comparison of correlation coefficients of dominant species at different altitudes.

Elevation	Correlation type	Distinctly significant (*p* ≤ .01)		Significant (*p* ≤ .05)		Not significant (*p* > .05)		Positive and negative correlation ratio
		Positive	Negative	Positive	Negative	Positive	Negative	
Totality	Pearson	1	0	7	1	15	81	0.28
	Spearman	1	0	1	3	46	54	0.84
N1	Pearson	3	0	2	0	15	85	0.24
	Spearman	3	0	6	2	21	73	0.40
N2	Pearson	4	0	5	0	11	85	0.24
	Spearman	9	0	4	3	12	77	0.35
N3	Pearson	4	0	1	1	13	86	0.21
	Spearman	6	2	4	2	26	65	0.52
N4	Pearson	4	0	2	0	17	82	0.28
	Spearman	2	0	3	0	24	66	0.44

*Note*: Elevation gradient: N1 (989–1091 m), N2 (1092–1193 m), N3 (1194–1295 m) and N4 (1296–1398 m).

The Spearman rank correlation test was employed to assess species covariance (Table 4, Figure 4). The findings corroborated those of the previous *χ* ^2^ and Pearson correlation tests, demonstrating that most species pairs within each community did not exhibit significant negative correlations. These results suggest that the dominant species within the community display a degree of randomness and a loose association, implying that the relationships between species are complex and that the community is likely in an early stage of ecological succession.

Analysis of totality Pearson's correlation coefficient and Spearman's rank correlation coefficient revealed significant positive correlations among two pairs of species: *Hyophila involuta* (S7) and *Bryum dichotomum* (S11), and *Didymodon ferrugineus* (S8) with *Eurohypnum leptothallum* (S12). These findings suggest that these closely associated species share similar biological traits and occupy overlapping ecological niches. We also found similar findings in different altitudinal gradients of N1–N4 (989–1398 m) (Table S3). Therefore, the dominant species of lithophytic moss have similar ecological habits, but their associations in different altitudinal gradients are quite different.

### 3.4 Analysis of community stability

**FIGURE 5:**
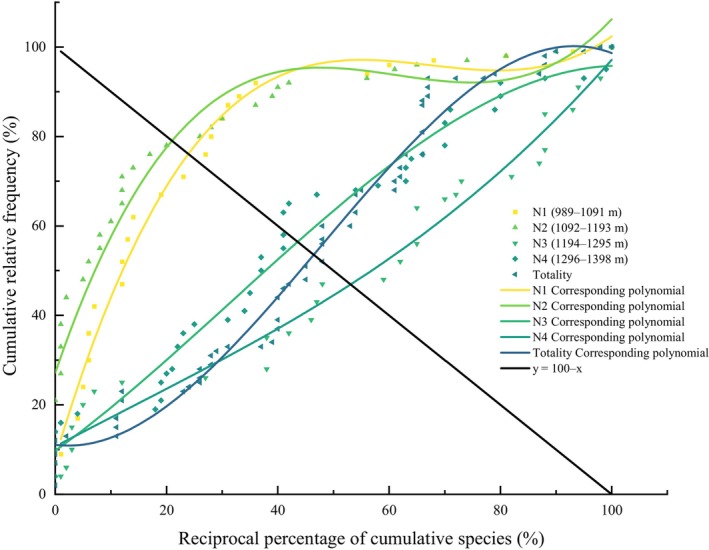
M. Godron stability fitting curve for lithophytic moss communities.

The stability of bryophyte communities across different altitudinal gradients was assessed using the M. Godron stability fitting curve (Figure 5) and its corresponding equation (Table 5). The results indicate a variation in community stability with altitude, in the following order: N2 (1092–1193 m) > N1 (989–1091 m) > N3 (1194–1295 m) > overall community > N4 (1296–1398 m). Notably, the N2 community, which closely approximates the stable intersection coordinate of 20/80, was in a relatively stable state, whereas the other altitudinal gradients exhibit instability.

**TABLE 5:** Results of stability analysis of lithophytic moss community.

Elevation	Fitting equation	Determination coefffcient (*R* ^2^)	Nodal coordinate	Distance of intersection point and stable point	Stability
Totality	*y* = −2.38116E‐4*x* ^3^ + 0.03415*x* ^2^ + 0.65834*x* + 11.07009	0.980	(46.45, 45.55)	19.89	Instability
N1	*y* = 3.38471E‐4*x* ^3^ − 0.06222*x* ^2^ + 3.62783*x* + 27.18635	0.942	(23.92, 76.08)	47.31	Instability
N2	*y* = 3.19676E‐4*x* ^3^ − 0.06456*x* ^2^ + 4.20152*x* + 8.13757	0.984	(20.97, 79.03)	15.33	Relatively stable
N3	*y* = −7.73216E‐5*x* ^3^ + 0.00734*x* ^2^ + 0.89954*x* + 9.70999	0.976	(43.56, 56.44)	21.05	Instability
N4	*y* = 3.42102E‐5*x* ^3^ − 0.00136*x* ^2^ + 0.65834*x* + 10.68453	0.975	(53.08, 46.92)	22.28	Instability

*Note*: Elevation gradient: N1 (989–1091 m), N2 (1092–1193 m), N3 (1194–1295 m) and N4 (1296–1398 m).

**FIGURE 6:**
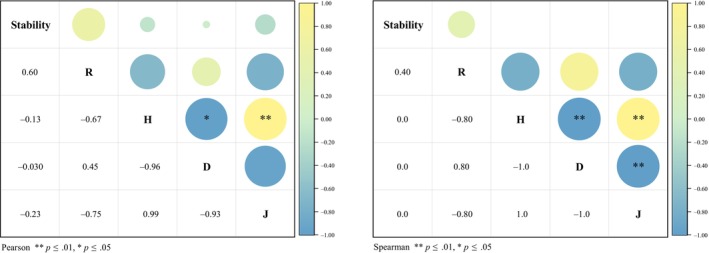
Correlation analysis of community stability and *α* diversity index of lithogenic bryophytes at different altitudes. R, Margalef richness index; H, Shannon–Wiener Diversity Index; D, Simpson dominance index; J, Pielou evenness index. *p ≤ .05, **p ≤ .01.

Correlation analyses were performed to examine the relationship between community stability and the *α* diversity index across four altitudinal gradients (Figure 6). The results indicated that neither the Pearson correlation coefficient nor the Spearman rank correlation coefficient revealed a significant association between community stability and species diversity (*p* > .05). However, a modest positive correlation was observed with the Margalef richness index. These findings suggest that the stability of lithophytic moss communities is not significantly influenced by species diversity across varying elevations.

### 3.5 Relationship between species composition, community stability, and environmental factors

**FIGURE 7:**
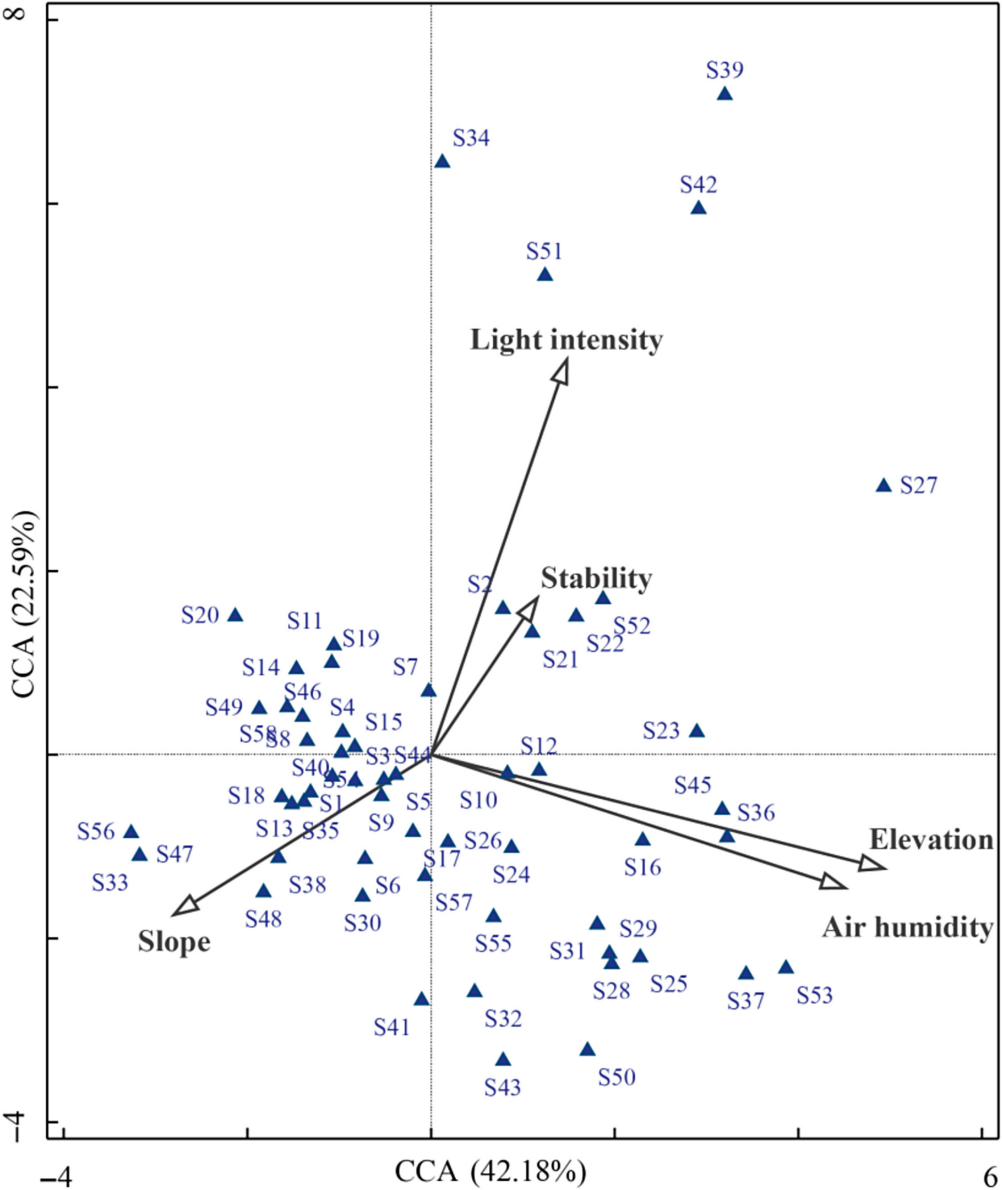
Typical correspondence analysis of lithophytic moss species with environmental factors and community stability (CCA). The species numbers for Si are shown in Table S1.

**FIGURE 8:**
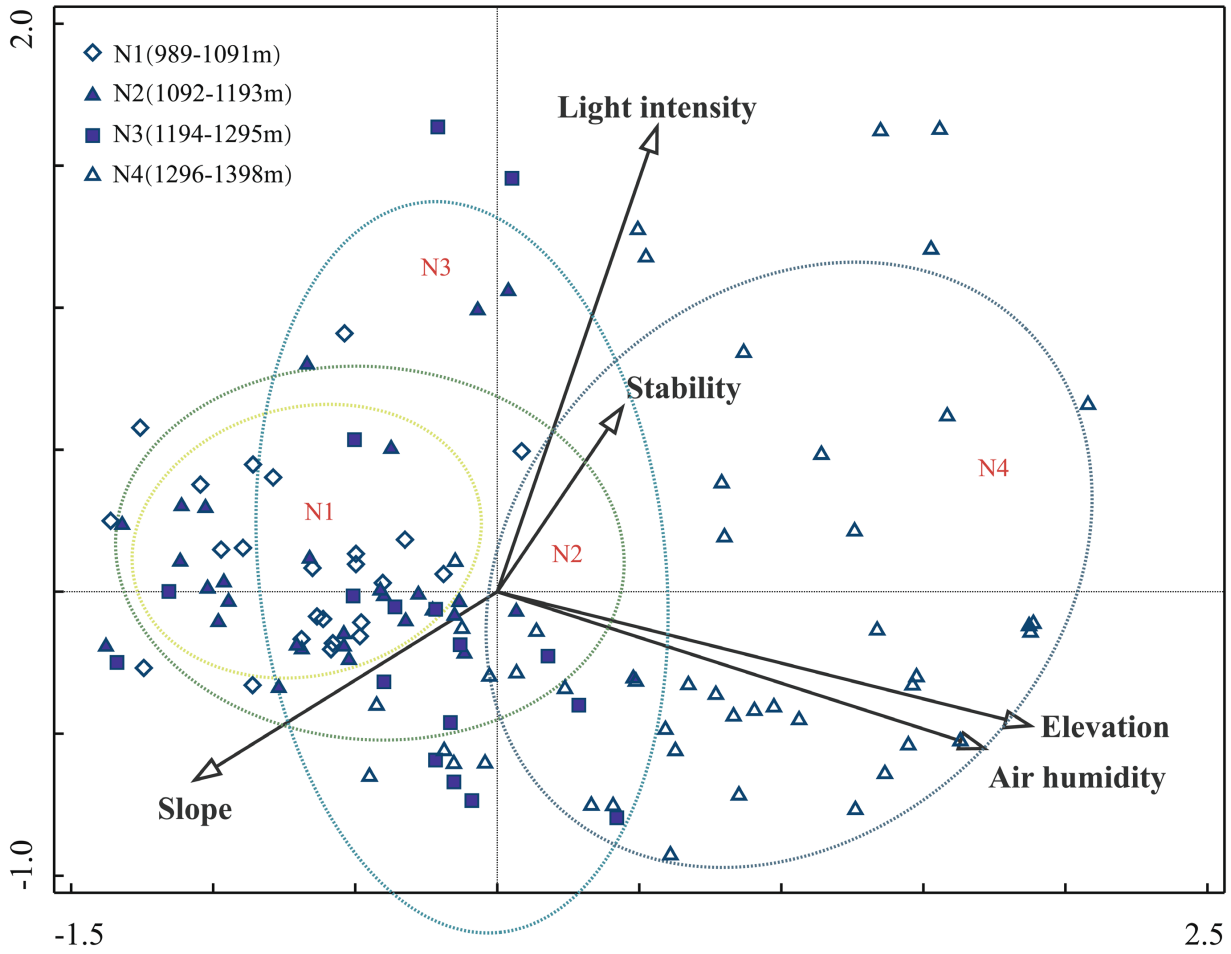
Typical correspondence analysis (CCA) of different elevation plots with environmental factors and community stability. Elevation gradient: N1 (989–1091 m), N2 (1092–1193 m), N3 (1194–1295 m) and N4 (1296–1398 m).

CCA was conducted to assess the relationship between species distribution and four significant environmental factors, as well as community stability, across 117 plots (Figures 7, 8; Tables S4, S5). The first two axes accounted for 64.77% of the variation in species distribution, suggesting that these axes effectively capture changes in species composition. Air humidity emerged as the most influential environmental factor (*p* = .002), followed by elevation (*p* = .002), light intensity (*p* = .030), and slope direction (*p* = .024), all of which exerted significant effects on species distribution (Table 6). Altitude and air humidity, which were positively correlated, had the strongest associations with the first CCA axis. Species such as *Brachythecium plumosum* (S16), *Thuidium kanedae* (S36), and *Hypnum hamulosum* (S45) were predominantly found at higher elevations (N4: 1296–1398 m) and aligned positively along this axis. Conversely, species from lower elevations (N1: 989–1193 m) were distributed in the opposite direction. The analysis revealed that increased air humidity correlated with a higher abundance of hygrophilous species, while species at lower altitudes tended to be more drought‐tolerant. Along the second CCA axis, a positive relationship was observed between light intensity and species distribution at mid‐elevation (N3: 1194–1295 m), with a corresponding decrease in slope gradient. Community stability was found to be negatively correlated with slope and positively correlated with light intensity. Species such as *Brachythecium salebrosum* (S2), *Didymodon tectorus* (S21), and *Timmiella anomala* (S52) showed a positive association with stability, indicating greater stability with increased light intensity.

**TABLE 6:** Explanation table of CCA environment variables.

Environmental factor	Explanatory quantity (%)	Contribution rate (%)	*p*
Air humidity	1.9	2.2	.002**
Elevation	1.9	2.2	.002**
Light intensity	1.4	1.6	.030*
Slope	1.2	1.4	.024*
Stability	1.3	1.5	.090

**p < .01, *p < .05.

In summary, air humidity and elevation are the primary environmental factors influencing species distribution, while community stability is most significantly affected by light intensity and is negatively associated with slope.

## 4 DISCUSSION

### 4.1 Composition and diversity of lithophytic moss

In the karst city under study, 58 lithophytic moss species from 14 families and 27 genera have been identified. The greatest species richness within these families occurs at the N4 altitudinal gradient (1296–1398 m), corroborating the findings of Jin Yalin (Jin & Wang, 2023), who reported a peak in lithophytic moss species at the altitude gradient III (1334–1515 m). The Brachytheciaceae and Pottiaceae families, which exhibit broad ecological ranges and adaptive mechanisms (Guo et al., 2017; Wang et al., 2018), are particularly dominant. Notably, the Pottiaceae family is characterized by its robust drought resistance, water retention, and soil conservation capabilities, making it prevalent in areas affected by rocky desertification (Cong et al., 2017, 2021). The dominant species are different at different altitudes, but they are all common species in karst areas. Research indicates that dominant species tend to be more stable than less prevalent ones and that increasing the relative abundance of such stable species can enhance community stability (Grman et al., 2010). Consequently, these dominant lithophytic moss species are valuable as pioneer species for vegetation ecological restoration in the region, playing a crucial role in expediting community succession and bolstering community stability.

Altitude is a critical factor influencing plant species diversity and composition (Wani et al., 2023). In the present study, the N4 altitudinal range (1296–1398 m) displayed the highest richness and dominance indices, whereas the N3 range (1194–1295 m) had the highest diversity and evenness indices. Previous research has identified a hump‐shaped pattern in bryophyte species richness along altitudinal gradients (Dani et al., 2023; Oishi, 2021), with the peak of plant diversity typically occurring at mid‐altitudes, where optimal temperature and precipitation conditions prevail (Manish, 2021). These conditions also foster ecological niches conducive to the settlement of diverse species (Doležal et al., 2023). Nonetheless, bryophyte species diversity is significantly influenced by altitude (Coelho et al., 2021). High‐altitude plant communities are found to be more susceptible to environmental stressors (Thakur & Chawla, 2019). However, bryophytes' distinctive morphological and physiological traits allow them to thrive in extreme conditions of drought, nutrient scarcity, and cold (Perera‐Castro et al., 2020; Yang et al., 2016). In the karst habitats examined, lithophytic moss species are prevalent across various elevations, particularly in the mid‐to‐high elevations of N3 (1194–1295 m) and N4 (1296–1398 m), where species richness and diversity are notably greater. This phenomenon may be attributed to the predominant distribution of study plots along the urban remnant mountain forest, characterized by high air humidity and forest canopy coverage, and reduced exposure of rocks to solar radiation. Additionally, the urbanization pace in the Baiyan and Guanshanhu districts is comparatively slower than in other areas, resulting in lower levels of human interference and thereby facilitating the colonization of the lithophytic moss community.

### 4.2 Analysis of interspecific association of lithophytic moss communities

Interspecific associations and correlations are indicative of community dynamics and species interactions (Hu et al., 2022). In this study, bryophyte communities across all elevations exhibited a significant negative overall association, with low interspecific correlation degrees and a minimal ratio of positive to negative correlations, suggesting that the community is in an early successional stage. Moreover, most dominant bryophyte species were ecologically distinct and spatially segregated, although some displayed strong interspecific associations, likely due to similar biological traits and niche overlap within the same genus (Lu et al., 2020). Correlation analyses revealed that most species pairs within the community did not exhibit significant negative correlations, implying that interspecific relationships were generally random and loose, and further confirmed that the community was in the early stage of unstable succession. Previous research posits that the interspecific relationships and community structure of bryophyte communities result from a combination of stochastic plant dispersal and habitat selection (Yuan et al., 2022). Lithophytic mosses, in particular, enhance the niche against rocky desertification through their biological characteristics (Cong et al., 2017; Zhang et al., 2023). Consequently, research on vegetation in karst regions should not only comprehend the ecological attributes of lithophytic moss in community reconstruction and restoration but also consider the impact of different habitats on interspecific relationships.

### 4.3 Analysis and environmental interpretation of interspecific stability of lithophytic moss communities

Community stability reflects the interconnectedness of species (Liu et al., 2017; Wang et al., 2022). Generally, lithophytic bryophyte communities across various elevations are in a state of flux, aligning with research on community associations and correlations. Notably, the stability coordinates in the N2 (1092–1193 m) elevation zone approximate Godron's stable point more closely. This may be attributed to the susceptibility of lower elevations to human disturbances and the adverse effects of low temperatures and suboptimal habitats at higher elevations on species’ roles within the plant community, thereby influencing community stability (Cabrera et al., 2019). Furthermore, the relationship between community diversity and stability remains contentious (Maestre et al., 2012; Wang et al., 2022). This study observed that lithophytic moss community stability did not markedly vary with species diversity, maintaining relative stability only at the N2 elevation with moderate diversity. These findings echo Xue et al.'s (2022) research, which also reported no definitive link between diversity and stability. This could suggest that species diversity fosters community stability only up to a certain threshold (Yu et al., 2015). Moreover, the intricate and ever‐changing urban habitats, impacted by factors, such as environmental pollution and human activity, significantly influence the distribution of lithophytic moss (Liu et al., 2015; Zhou et al., 2021). Consequently, the relationship between community diversity and stability is rendered ambiguous. The dynamic nature of this relationship and the interactions therein warrant further investigation.

Recent studies have posited that both the traits of species within a community and environmental factors can influence community stability (Xue et al., 2022). In this research, light intensity was found to significantly impact the stability of bryophyte communities, exhibiting a negative correlation with slope. Species, such as *Brachythecium*, *Didymodon*, and *Bryum* showed a positive correlation with stability, suggesting their high tolerance to light and prevalence in well‐lit areas (Jagodziński et al., 2018). Drought‐resistant mosses like *Didymodon* and *Bryum* may act as pioneer species in community succession due to their specialized leaf surfaces and dense rhizoid structures, which confer strong soil stabilization and adsorption capabilities, playing a crucial role in the ecological restoration of rocky desertification (Tu et al., 2021). Furthermore, air humidity and elevation emerged as primary environmental factors influencing the distribution of lithophytic moss species in the region, corroborating Duan's findings (Duan & Wang, 2023). Elevation, as an environmental gradient, significantly affects the distribution of saxicolous bryophytes, reflecting the intricate variations in ecological parameters, such as rainfall, temperature, humidity, and solar radiation across communities (Araujo et al., 2022). The majority of sampling sites in this study were situated in karst geological areas characterized by sparse vegetation and intense light exposure. The growth of lithophytic moss on rock surfaces can ameliorate the microclimatic conditions of the substrate and absorb atmospheric moisture (García et al., 2016), thereby influencing the distribution and structure of bryophyte communities. In conclusion, the distribution and stability of the lithophytic moss community are closely related to its characteristics and environmental factors.

Understanding the community structure, interspecific relationships, and stability of urban lithophytic moss in karst environments is crucial for ecological restoration and community development. However, the formation of interspecific associations is influenced by numerous complex factors, and the underlying causes remain elusive. Further research into the internal mechanisms of bryophyte interspecific relationships is warranted, incorporating plant physiology, genetics, and molecular ecology. Additionally, community succession is a protracted dynamic process that necessitates long‐term environmental monitoring to yield precise interpretations of plant community characteristics and stability in karst regions and analogous areas. Based on this study's findings, it is advisable to select dominant lithophytic moss species with robust environmental adaptability and positive interspecific correlations for collaborative planting at appropriate altitudinal zones. This strategy aims to enhance community structure, mitigate detrimental interspecies competition, and foster vegetation restoration and community stability in karst urban settings.

## 5 CONCLUSIONS

In this study, 58 lithophytic moss species from 27 genera and 13 families were identified across four altitudinal zones (N1–N4, ranging from 989 to 1398 m) in a karst city. The most prevalent families were Brachytheciaceae and Pottiaceae. N4 (1296–1398 m) exhibited the highest species richness and dominance values, while N3 (1194–1295 m) showed the highest species diversity and uniformity values, with N2 (1092–1193 m) displaying intermediate values. The overall association of the lithophytic moss community was significantly negative across all altitude gradients, indicating that the community is generally in the primary stage of succession. Most advantages within the community are not strongly associated with each other and tend to be relatively independent. The stability analysis using the M. Godron method further revealed that except for N2 (1092–1193 m), the other elevations were unstable, and the community's stability did not vary with the level of species diversity. Additionally, light intensity (182–129300 lux) exerted the greatest influence on community stability, while air humidity (36.5–52.3%) and altitude (998‐1327 m) were identified as the primary environmental factors affecting the distribution of lithophytic moss species. These research findings will provide fundamental data and scientific guidance for restoring plant ecology in urban areas.

## AUTHOR CONTRIBUTIONS

**Lixin Duan:** Conceptualization (equal); data curation (equal); investigation (equal); methodology (equal); resources (equal); visualization (lead); writing – original draft (lead); writing – review and editing (equal). **Xiurong Wang:** Conceptualization (equal); supervision (equal); writing – review and editing (lead). **Yalin Jin:** Conceptualization (equal); investigation (equal); methodology (equal); resources (equal).

## CONFLICT OF INTEREST STATEMENT

The authors declare no conflict of interest.

## Supporting information


Table S1.

Table S6.

## Data Availability

All data is provided in the public data repository Dryad and support information. 10.5061/dryad.k0p2ngfff. Reviewer URL: https://datadryad.org/stash/share/UjV0da6lwq7bhrQ5Sg2EhCGvlPvb1WYjgnUeKgb5XFM.
